# Crystal structures of 2-bromo-1,1,1,3,3,3-hexa­methyl-2-(tri­methyl­sil­yl)tris­ilane and 2-bromo-1,1,1,3,3,3-hexa­isopropyl-2-(triiso­propyl­sil­yl)tri­silane

**DOI:** 10.1107/S2056989018009696

**Published:** 2018-07-24

**Authors:** Eva M. Gulotty, Richard J. Staples, Shannon M. Biros, Peter P. Gaspar, Nigam P. Rath, William R. Winchester

**Affiliations:** a Department of Chemistry, Grand Valley State University, 1 Campus Dr., Allendale, MI 49401, USA; b Center for Crystallographic Research, Department of Chemistry, Michigan State, University, 578 S. Shaw Lane, East Lansing, MI, USA; c Department of Chemistry, Washington University, St. Louis, MO 63130-4899, USA; d Department of Chemistry, University of Missouri St. Louis, St. Louis, MO 63121-4499, USA

**Keywords:** crystal structure, HypSiBr, TipSiBr, supersilyl bromide, steric hindrance, intra­molecular C-H⋯Br hydrogen bonding

## Abstract

The synthesis and crystal structures of two tris­(tri­alkyl­sil­yl)silyl bromide compounds, C_9_H_27_BrSi_4_ (**I**, HypSiBr) and C_27_H_63_BrSi_4_ (**II**, TipSiBr), are described. Compound **I** was prepared in 85% yield by free-radical bromination of 1,1,1,3,3,3-hexa­methyl-2-(tri­methyl­sil­yl)tris­ilane using bromo­butane and 2,2′-azobis(2-methyl­propio­nitrile) as a radical initiator at 333 K. The mol­ecule possesses threefold rotational symmetry, with the central Si atom and the Br atom being located on the threefold rotation axis. The Si—Br bond distance is 2.2990 (12) Å and the Si—Si bond lengths are 2.3477 (8) Å. The Br—Si—Si bond angles are 104.83 (3)° and the Si—Si—Si bond angles are 113.69 (2)°, reflecting the steric hindrance inherent in the three tri­methyl­silyl groups attached to the central Si atom. Compound **II** was prepared in 55% yield by free-radical bromination of 1,1,1,3,3,3-hexa­isopropyl-2-(triiso­propyl­sil­yl)tris­ilane using *N*-bromo­succinimide and 2,2′-azobis(2-methyl­propio­nitrile) as a radical initiator at 353 K. Here the Si—Br bond length is 2.3185 (7) Å and the Si—Si bond lengths range from 2.443 (1) to 2.4628 (9) Å. The Br—Si—Si bond angles range from 98.44 (3) to 103.77 (3)°, indicating steric hindrance between the three triiso­propyl­silyl groups.

## Abstract 2

The title compounds, **I** (HypSiBr), and **II** (TipSiBr), crystallized in the cubic space group *Pa* 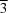 and the triclinic space group *P* 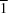, respectively. In both structures the central silicon atom is sterically hindered with bond angles that deviate from the expected 109.5° of a tetra­hedron.

## Chemical context   

The steric and electronic effects of the tris­(tri­methyl­sil­yl)silane group have been exploited for the synthesis and study of a variety of reactive centers including silylenes (Wendel *et al.*, 2017 ▸) and silylanions (Kayser *et al.*, 2002 ▸; Mechtler *et al.*, 2004 ▸; Zirngast *et al.*, 2008 ▸; Marschner, 2015 ▸). This sterically hindered group has been shown to lead to lower coordination by solvent when it is attached to organolithium compounds (Feil & Harder, 2003 ▸). It has also been used in organic synthesis to produce highly stereoselective aldol reactions leading to unique reactivity (Gati & Yamamoto, 2016 ▸). For this research we prepared tris­(tri­methyl­sil­yl)silyl­bromide (HypSiBr) as a precursor to vinyl­tris­(tri­methyl­sil­yl)silane. The even bulkier tris­(triiso­propyl­sil­yl)silylbromide (TipSiBr) was prepared as a potential precursor to meth­oxy­tris­(triiso­propyl­sil­yl)silane. Herein, we report on the crystal structures of these two sterically hindered silylbromides 2-bromo-1,1,1,3,3,3-hexa­methyl-2-(tri­methyl­sil­yl)tris­ilane (**I**), and 2-bromo-1,1,1,3,3,3-hexa­isopropyl-2-(triiso­propyl­sil­yl)tris­ilane (**II**).
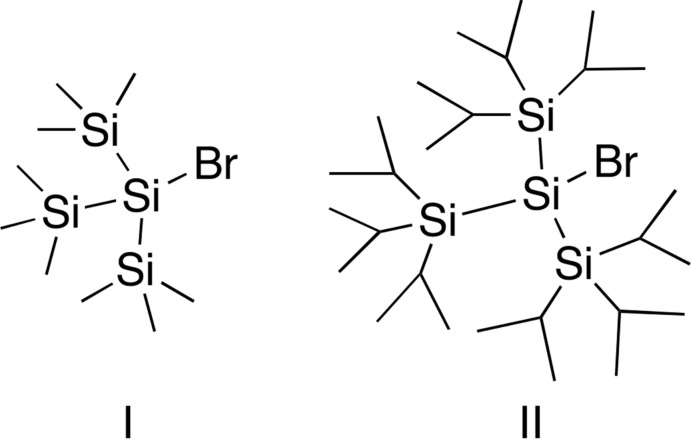


## Structural commentary   

**Figure 1:**
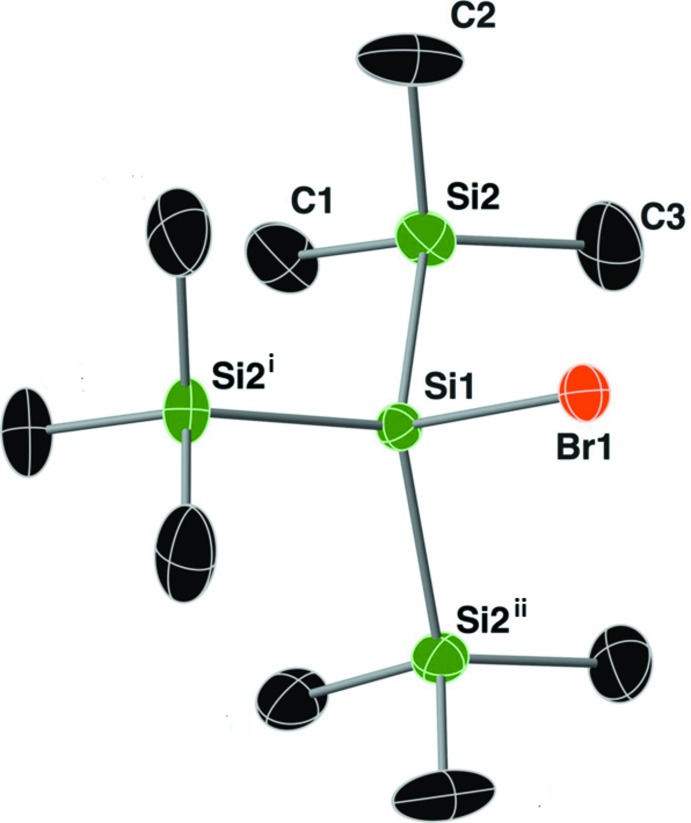
A view of the mol­ecular structure of compound **I**, with atom labelling. Displacement ellipsoids are drawn at the 30% probability level. Hydrogen atoms have been omitted for clarity. Unlabelled atoms are related to the labelled atoms by threefold rotation symmetry [symmetry codes: (i) *z*, *x*, *y*; (ii) *y*, *z*, *x*].

The mol­ecular structure of compound **I** (HypSiBr), is shown in Fig. 1 ▸, and selected geometrical parameters are given in Table 1 ▸. The asymmetric unit is composed of one tri­methyl­silyl group, with the central silicon atom Si1 and the bromine atom Br1 lying on a threefold rotation axis. This supersilylbromide crystallized in the cubic space group *Pa* 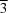 with a central 4-coordinate silicon atom, Si1, that deviates slightly from an ideal tetra­hedron due to the steric bulk of the attached tri­methyl­silyl (TMS) groups. The τ_4_ descriptor for fourfold coordination around Si1 is 0.94 (where, for extreme forms, τ_4_ = 0.00 for square-planar, 1.00 for tetra­hedral and 0.85 for trigonal–pyramidal; Yang *et al.*, 2007 ▸). Inter­estingly, the τ_4_ descriptor for fourfold coordination around the TMS atom Si2 is 0.99, which demonstrates an ideal tetra­hedral geometry around this silicon atom. The Si2—Si1—Si2^i,ii^ bond angle is 113.69 (2)° while the Br1—Si1—Si2 bond angle is 104.83 (3)°, indicating that the tri­methyl­silyl groups are forced away from one another. The Si1—Br1 bond length is 2.2990 (12) Å. As for Si2, the C—Si2—C bond angles range from 107.1 (2) to 110.55 (17)°, while the C—Si2—Si1 bond angles range from 108.61 (10) to 110.16 (11)°.

**Figure 2:**
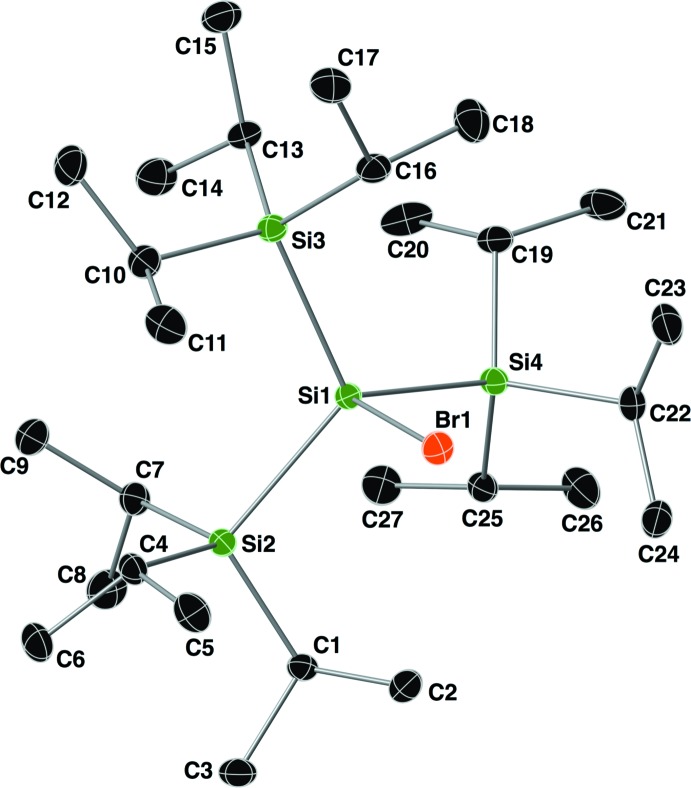
A view of the mol­ecular structure of compound **II**, with atom labelling. Displacement ellipsoids are drawn at the 30% probability level, and all hydrogen atoms have been omitted for clarity.

The asymmetric unit of compound **II** (TipSiBr), is shown in Fig. 2 ▸, and selected geometrical parameters are given in Table 1 ▸. This compound crystallized in the triclinic space group *P* 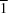 with a central four-coordinate silicon atom, Si1, that deviates from the ideal tetra­hedron as shown from its τ_4_ descriptor for fourfold coordination of 0.90. The Br1—Si1—Si2/Si3/Si4 bond angles range from 98.44 (3) to 103.77 (3)°, and the Si1—Br1 bond distance is 2.3185 (7) Å, which is longer than that of compound **I** [2.2990 (12) Å]. The τ_4_ descriptor values for atoms Si2, Si3 and Si4 (the silicon atoms of the triiso­propyl­silyl groups) are 0.96, 0.97 and 0.95, respectively, indicating that their coordination geometry is closest to an ideal tetra­hedron.

## Supra­molecular features   

**Figure 3:**
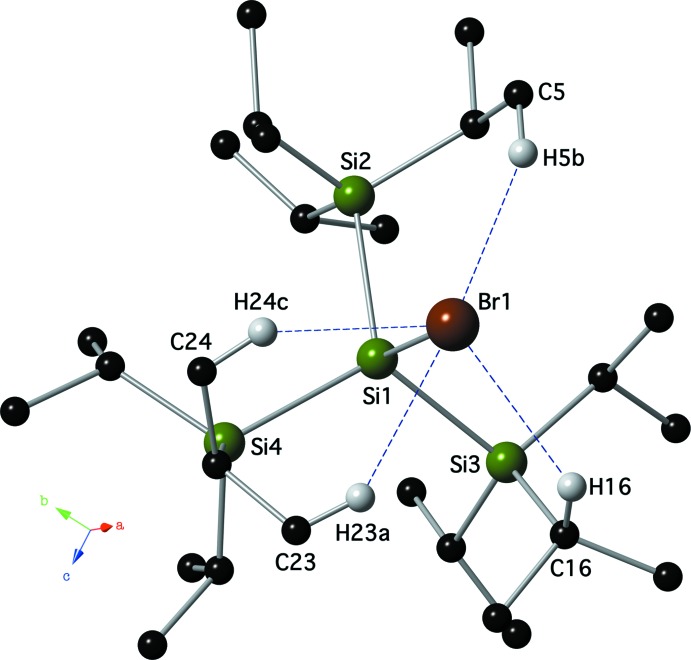
Intra­molecular C—H⋯Br hydrogen bonds (blue dashed lines; see Table 2 ▸) present in compound **II**. For clarity, only the hydrogen atoms involved in a hydrogen bonding are shown.

There are no significant inter­molecular contacts, other than weak van der Waals inter­actions, present in the crystals of compounds **I** or **II**. Compound **II**, however, contains four intra­molecular C—H⋯Br hydrogen bonds (Table 2 ▸, Fig. 3 ▸). These hydrogen bonds contain *D*⋯*A* distances that range from from 3.584 (3) to 3.726 (3) Å, and *D*—H⋯*A* bond angles that range from 131 to 155°.

## Database survey   

The Cambridge Structural Database (CSD, version 5.39, February 2018; Groom *et al.*, 2016 ▸) contains 1398 structures containing a Si_3_Si group. Of these, there are only 42 structures where the central silicon atom is bonded directly to a halogen.

Of particular inter­est to this work is the structure of tris(tri­methyl­sil­yl)chloro­silane (**III**, HypSiCl) [CSD refcode QULWEA; Kuzora *et al.*, 2009 ▸], the isotypic chloro derivative of compound **I**, and the structure of (iPr_3_Si)_3_SiH (**IV**, TipSiH), isotypic with compounds **I** and **III**. The analysis of **IV** by both X-ray and neutron diffraction has been described by Gaspar *et al.* (1999 ▸). Table 1 ▸ contains pertinent bond lengths and bond angles for compounds **I**, **II**, **III** (HypSiCl) and **IV** (TipSiH).

For compounds **I** and **III** the Si—*X* bond lengths follow the expected trend with the Si1—Cl bond length of QULWEA at 2.1248 (9) Å compared to the Si1—Br1 bond length of 2.2990 (12) Å for compound **I**. The Si1—Si2 bond length of the bromo derivative **I** reported here is 2.3477 (8) Å, which is slightly longer than the Si1—Si2 bond length of the chloro derivative at 2.3406 (6) Å. The central silicon atom of the chloro derivative appears less sterically hindered with an Si2—Si1—Cl1 bond angle of 105.508 (18)° and Si2—Si1—Si2^i,ii^ bond angles of 113.126°, *versus* a smaller Si2—Si1—Br1 bond angle of 104.83 (3)° and a larger Si2—Si1—Si2^i,ii^ bond angle of 113.69 (2)° for compound **I** [symmetry codes: (i) *z*, *x*, y; (ii) *y*, *z*, *x*]. The protio derivative (HypSiH) is a liquid at room temperature, and the structure of the iodo derivative (HypSiI) has not been deposited in the CSD.

The X-ray data for compound **IV** (TipSiH) was not found in the CSD, but the journal article (Gaspar *et al.*, 1999 ▸) contains all pertinent structural data to allow for a comparison with (iPr_3_Si)_3_SiBr, *viz*. compound **II** (TipSiBr). Like compounds **I** and **III**, compound **IV** crystallizes in the cubic space group *Pa* 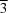, and the mol­ecule possesses threefold rotation symmetry. The presence of a small hydrogen atom bonded to the central silicon atom Si1 allows the three (^*i*^Pr_3_)Si– groups to push further away from one another, resulting in Si2—Si1—Si2^i,ii^ bond angles of 117.9 (1)° and Si2^i,ii^—Si1—H bond angles of 98.3 (1)° [symmetry codes: (i) *z*, *x*, *y*; (ii) *y*, *z*, *x*]. In **II**, the corresponding Si—Si—Si bond angles range from 115.02 (4) to 116.59 (4)° and the Si—Si—Br bond angles vary from 98.44 (3) to 103.77 (3)°.

## Synthesis and crystallization   

**Compound I:** Tris(tri­methyl­sil­yl)silane (2.0 g, 8.0 mmol) was added to an oven-dried nitro­gen-flushed 250 ml Schlenk flask with a stir-bar. Bromo­butane (2.0 g, 14.6 mmol) was filtered through a plug of silica gel in a Pasteur pipette and was transferred into the Schlenk flask. AIBN [2,2-azobis(2-methyl­propio­nitrile); 20 mg] was then added to the flask, and the reaction was heated to 333 K using an oil bath and then heating was stopped. After stirring the reaction overnight at room temperature, GC–MS analysis of a sample indicated incomplete reaction and more AIBN (11 mg) was added to the flask. The reaction was heated once more to 333 K for 1 h. Analysis by GC–MS now indicated that the reaction was complete. The flask was placed in a freezer at 243 K and colourless block-like crystals of **I** formed overnight. Removal of the solvent *in vacuo* yielded 2.2 g (85%). ^1^H NMR (300 MHz, chloro­form-*d*) δ 0.24 (*s*, 27H); ^13^C NMR (75 MHz, chloro­form-*d*) δ −0.51 ppm; GC–MS: 11.24 min, *m*/*z* = 328, base peak: 73.

**Compound II:** Tris(triiso­propyl­sil­yl)silane (110 mg, 0.22 mmol) was dissolved in freshly distilled benzene (10 ml) along with NBS (45 mg) and AIBN (2 mg, initiator). The mixture was heated using an oil bath at 333 K for 30 min, when GC–MS analysis indicated that no reaction had occurred. At this point the solution was heated with a heat gun until the reaction mixture turned slightly yellow. The yellow colour dissipated in less than 1 min. Analysis of the reaction mixture by ^1^H NMR indicated that only 60% of the starting material had been consumed. An additional amount of NBS (*N*-bromo­succinimide; 20 mg) was added to the reaction flask, and the solution was again heated with a heat gun. The product was isolated by removing the solvent *in vacuo* and extracting the product from the crude reaction mixture with pentane. The pentane solution was filtered through glass wool, concentrated and weighed (135 mg). Analysis of the product with ^1^H NMR indicated this was 90% pure. The product was further purified by dissolving this solid in 1 ml pentane, cooling to 195 K and isolating the colourless needle-like crystals of **II** by removing the solvent with a syringe, washing with pentane and drying *in vacuo* (yield 62 mg, 55%). ^1^H NMR (300 MHz, C_6_D_6_) δ 1.34 (*d*, *J* = 7.3 Hz, 54H), 1.66 (*heptet*, *J* = 7.4 Hz, 9H); ^13^C NMR (75 MHz, chloro­form-*d*) δ 16.4, 21.6; HRMS for C_17_H_63_BrSi_4_ calculated 535.2642 (*M* − C_3_H_7_), found 535.2641.

## Refinement   

Crystal data, data collection and structure refinement details are summarized in Table 3 ▸. For both compounds the hydrogen atoms were placed in calculated positions and refined using a riding model: C—H = 0.98-1.00 Å with *U*_iso_(H) = 1.5*U*_eq_(C-meth­yl) and 1.2*U*_eq_(C) for other H atoms.

## Supplementary Material

Crystal structure: contains datablock(s) I, II, Global. DOI: 10.1107/S2056989018009696/su5451sup1.cif

Structure factors: contains datablock(s) I. DOI: 10.1107/S2056989018009696/su5451Isup2.hkl

Structure factors: contains datablock(s) II. DOI: 10.1107/S2056989018009696/su5451IIsup3.hkl

Supporting information file. DOI: 10.1107/S2056989018009696/su5451Isup4.cml

Supporting information file. DOI: 10.1107/S2056989018009696/su5451IIsup5.cml

CCDC references: 1854536, 1854535

Additional supporting information:  crystallographic information; 3D view; checkCIF report

## Figures and Tables

**Table 1:** Selected bond lengths (Å), bond angles (°) and the fourfold coordination descriptor, τ_4_,^*a*^ for compounds **I** (HypSiBr), **II** (TipSiBr), **III** (HypSiCl) and **IV** (TipSiH)

Compound	**I** (HypSiBr)	**II** (TipSiBr)	**III** (HypSiCl)^*b*^	**IV** (TipSiH)^*c*^
Si1—*X* ^*d*^	2.2990 (12)	2.3185 (7)	2.1248 (9)	1.608 (1)
Si1—Si*n* ^*e*^	2.3477 (8)	2.4430 (10), 2.4448 (10), 2.4628 (9)	2.3406 (6)	2.405 (1)
Si2—Si1—Si*n* ^*f*^	113.69 (2)	115.02 (4), 116.55 (3), 116.59 (4)	113.13 (2)	117.9 (1)
Si2—Si1—*X* ^*d*^	104.83 (3)	98.44 (3) to 103.77 (3)	105.51 (2)	98.3 (1)
τ_4_ of Si1	0.94	0.90	0.95	0.88

Notes: (*a*) Yang *et al.* (2007 ▸); (*b*) Kuzora *et al.* (2009 ▸); (*c*) X-ray data (Gaspar *et al.*, 1999 ▸); (*d*) *X* = Br for **I** and **II**, Cl for **III**, and H for **IV**; (*e*) *n* = 2 for **I**, **III** and **IV**, and 2, 3 and 4 for **II**; (*f*) *n* = 2^i^ and 2^ii^ for **I**, **III** and **IV** [symmetry codes: (i) = *z*, *x*, *y*; (ii) = *y*, *z*, *x*], and 2, 3 and 4 for **II**.

**Table 2:** Hydrogen-bond geometry (Å, °) for **II** [Chem scheme1]

*D*—H⋯*A*	*D*—H	H⋯*A*	*D*⋯*A*	*D*—H⋯*A*
C5—H5*B*⋯Br1	0.98	2.80	3.711 (3)	155
C16—H16⋯Br1	1.00	2.84	3.584 (3)	131
C23—H23*A*⋯Br1	0.98	2.87	3.685 (3)	142
C24—H24*C*⋯Br1	0.98	2.93	3.726 (3)	139

**Table 3:** Experimental details

	**I**	**II**
Crystal data		
Chemical formula	C_9_H_27_BrSi_4_	C_27_H_63_BrSi_4_
*M*_r_	327.57	580.04
Crystal system, space group	Cubic, *P* *a* 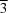	Triclinic, *P* 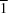
Temperature (K)	173	173
*a*, *b*, *c* (Å)	15.6497 (19), 15.6497 (19), 15.6497 (19)	8.4412 (4), 11.1336 (6), 18.8477 (10)
α, β, γ (°)	90, 90, 90	92.565 (4), 90.527 (4), 108.718 (4)
*V* (Å^3^)	3832.8 (14)	1675.44 (15)
*Z*	8	2
Radiation type	Mo *K*α	Mo *K*α
μ (mm^−1^)	2.37	1.38
Crystal size (mm)	0.45 × 0.24 × 0.14	0.38 × 0.10 × 0.02

Data collection		
Diffractometer	Bruker APEXII CCD	Bruker SMART APEX CCD area detector
Absorption correction	Multi-scan (*SADABS*; Bruker, 2014 ▸)	Multi-scan (*SADABS*; Bruker, 2014 ▸)
*T*_min_, *T*_max_	0.571, 0.745	0.554, 0.674
No. of measured, independent and observed [*I* > 2σ(*I*)] reflections	11038, 1174, 953	21505, 6628, 4752
*R*_int_	0.046	0.057
(sin θ/λ)_max_ (Å^−1^)	0.601	0.622

Refinement		
*R*[*F* ^2^ > 2σ(*F* ^2^)], *wR*(*F* ^2^), *S*	0.028, 0.075, 1.05	0.037, 0.075, 1.01
No. of reflections	1174	6628
No. of parameters	46	307
H-atom treatment	H-atom parameters constrained	H-atom parameters constrained
Δρ_max_, Δρ_min_ (e Å^−3^)	0.31, −0.16	0.36, −0.28

Computer programs: *APEX2*, *SMART* and *SAINT* (Bruker, 2014 ▸), *SHELXS97* and *SHELXTL* (Sheldrick, 2008 ▸), *SHELXT2013* (Sheldrick, 2015*a* ▸), *SHELXL2017* (Sheldrick, 2015*b* ▸), *OLEX2* (Dolomanov *et al.*, 2009 ▸; Bourhis *et al.*, 2015 ▸) and *CrystalMaker* (Palmer, 2007 ▸).

## supplementary crystallographic information

### 2-Bromo-1,1,1,3,3,3-hexamethyl-2-(trimethylsilyl)trisilane (I) Crystal data

**Table:**

C_9_H_27_BrSi_4_	Mo *K*α radiation, λ = 0.71073 Å
*M**_r_* = 327.57	Cell parameters from 3863 reflections
Cubic, *P**a*3	θ = 2.3–25.3°
*a* = 15.6497 (19) Å	µ = 2.37 mm^−^^1^
*V* = 3832.8 (14) Å^3^	*T* = 173 K
*Z* = 8	Block, colourless
*F*(000) = 1376	0.45 × 0.24 × 0.14 mm
*D*_x_ = 1.135 Mg m^−^^3^	

### 2-Bromo-1,1,1,3,3,3-hexamethyl-2-(trimethylsilyl)trisilane (I) Data collection

**Table:**

Bruker APEXII CCD diffractometer	953 reflections with *I* > 2σ(*I*)
φ and ω scans	*R*_int_ = 0.046
Absorption correction: multi-scan (SADABS; Bruker, 2014)	θ_max_ = 25.3°, θ_min_ = 2.3°
*T*_min_ = 0.571, *T*_max_ = 0.745	*h* = −18→16
11038 measured reflections	*k* = −18→17
1174 independent reflections	*l* = −15→18

### 2-Bromo-1,1,1,3,3,3-hexamethyl-2-(trimethylsilyl)trisilane (I) Refinement

**Table:**

Refinement on *F*^2^	Primary atom site location: structure-invariant direct methods
Least-squares matrix: full	Secondary atom site location: difference Fourier map
*R*[*F*^2^ > 2σ(*F*^2^)] = 0.028	Hydrogen site location: inferred from neighbouring sites
*wR*(*F*^2^) = 0.075	H-atom parameters constrained
*S* = 1.05	*w* = 1/[σ^2^(*F*_o_^2^) + (0.0349*P*)^2^ + 1.4333*P*] where *P* = (*F*_o_^2^ + 2*F*_c_^2^)/3
1174 reflections	(Δ/σ)_max_ = 0.002
46 parameters	Δρ_max_ = 0.31 e Å^−^^3^
0 restraints	Δρ_min_ = −0.16 e Å^−^^3^

### 2-Bromo-1,1,1,3,3,3-hexamethyl-2-(trimethylsilyl)trisilane (I) Special details

**Table:**

Geometry. All esds (except the esd in the dihedral angle between two l.s. planes) are estimated using the full covariance matrix. The cell esds are taken into account individually in the estimation of esds in distances, angles and torsion angles; correlations between esds in cell parameters are only used when they are defined by crystal symmetry. An approximate (isotropic) treatment of cell esds is used for estimating esds involving l.s. planes.

### 2-Bromo-1,1,1,3,3,3-hexamethyl-2-(trimethylsilyl)trisilane (I) Fractional atomic coordinates and isotropic or equivalent isotropic displacement parameters (Å^2^)

**Table:**

	*x*	*y*	*z*	*U*_iso_*/*U*_eq_	
Br1	0.74011 (2)	0.74011 (2)	0.74011 (2)	0.04337 (17)	
Si1	0.65529 (4)	0.65529 (4)	0.65529 (4)	0.0327 (3)	
Si2	0.69238 (5)	0.51472 (4)	0.69229 (5)	0.0473 (2)	
C1	0.6249 (2)	0.43945 (18)	0.6286 (2)	0.0734 (10)	
H1A	0.6331	0.4506	0.5675	0.110*	
H1B	0.5646	0.4478	0.6434	0.110*	
H1C	0.6415	0.3805	0.6414	0.110*	
C2	0.8079 (2)	0.49575 (19)	0.6707 (3)	0.0943 (14)	
H2A	0.8227	0.4370	0.6866	0.141*	
H2B	0.8422	0.5358	0.7044	0.141*	
H2C	0.8194	0.5044	0.6098	0.141*	
C3	0.6752 (3)	0.49746 (19)	0.8090 (2)	0.0953 (13)	
H3A	0.6142	0.5032	0.8222	0.143*	
H3B	0.7076	0.5401	0.8414	0.143*	
H3C	0.6946	0.4401	0.8247	0.143*	

### 2-Bromo-1,1,1,3,3,3-hexamethyl-2-(trimethylsilyl)trisilane (I) Atomic displacement parameters (Å^2^)

**Table:**

	*U*^11^	*U*^22^	*U*^33^	*U*^12^	*U*^13^	*U*^23^
Br1	0.04337 (17)	0.04337 (17)	0.04337 (17)	−0.00621 (10)	−0.00621 (10)	−0.00621 (10)
Si1	0.0327 (3)	0.0327 (3)	0.0327 (3)	−0.0008 (3)	−0.0008 (3)	−0.0008 (3)
Si2	0.0529 (5)	0.0317 (4)	0.0573 (5)	−0.0013 (3)	−0.0106 (4)	0.0008 (3)
C1	0.087 (2)	0.0426 (16)	0.091 (2)	−0.0092 (16)	−0.0304 (19)	−0.0051 (16)
C2	0.056 (2)	0.0494 (19)	0.178 (4)	0.0145 (16)	−0.007 (2)	0.001 (2)
C3	0.168 (4)	0.052 (2)	0.066 (2)	0.006 (2)	−0.019 (2)	0.0183 (17)

### 2-Bromo-1,1,1,3,3,3-hexamethyl-2-(trimethylsilyl)trisilane (I) Geometric parameters (Å, º)

**Table:**

Br1—Si1	2.2990 (12)	Si2—C1	1.870 (3)
Si1—Si2^i^	2.3478 (8)	Si2—C2	1.862 (3)
Si1—Si2	2.3477 (8)	Si2—C3	1.866 (3)
Si1—Si2^ii^	2.3478 (8)		
			
Br1—Si1—Si2	104.83 (3)	C1—Si2—Si1	108.61 (10)
Br1—Si1—Si2^ii^	104.83 (3)	C2—Si2—Si1	110.16 (11)
Br1—Si1—Si2^i^	104.83 (3)	C2—Si2—C1	110.55 (17)
Si2—Si1—Si2^ii^	113.69 (2)	C2—Si2—C3	107.12 (19)
Si2—Si1—Si2^i^	113.69 (3)	C3—Si2—Si1	109.96 (10)
Si2^i^—Si1—Si2^ii^	113.69 (2)	C3—Si2—C1	110.45 (16)

Symmetry codes: (i) *y*, *z*, *x*; (ii) *z*, *x*, *y*.

### 2-Bromo-1,1,1,3,3,3-hexaisopropyl-2-(triisopropylsilyl)trisilane (II) Crystal data

**Table:**

C_27_H_63_BrSi_4_	*Z* = 2
*M**_r_* = 580.04	*F*(000) = 632
Triclinic, *P*1	*D*_x_ = 1.150 Mg m^−^^3^
*a* = 8.4412 (4) Å	Mo *K*α radiation, λ = 0.71073 Å
*b* = 11.1336 (6) Å	Cell parameters from 8192 reflections
*c* = 18.8477 (10) Å	θ = 2.0–26.0°
α = 92.565 (4)°	µ = 1.38 mm^−^^1^
β = 90.527 (4)°	*T* = 173 K
γ = 108.718 (4)°	Needles, colourless
*V* = 1675.44 (15) Å^3^	0.38 × 0.10 × 0.02 mm

### 2-Bromo-1,1,1,3,3,3-hexaisopropyl-2-(triisopropylsilyl)trisilane (II) Data collection

**Table:**

Bruker SMART APEX CCD area detector diffractometer	6628 independent reflections
Radiation source: sealed tube	4752 reflections with *I* > 2σ(*I*)
Detector resolution: 8 pixels mm^-1^	*R*_int_ = 0.057
ω and φ scans	θ_max_ = 26.3°, θ_min_ = 1.1°
Absorption correction: multi-scan (SADABS; Bruker, 2014)	*h* = −10→10
*T*_min_ = 0.554, *T*_max_ = 0.674	*k* = −13→13
21505 measured reflections	*l* = −23→23

### 2-Bromo-1,1,1,3,3,3-hexaisopropyl-2-(triisopropylsilyl)trisilane (II) Refinement

**Table:**

Refinement on *F*^2^	Primary atom site location: structure-invariant direct methods
Least-squares matrix: full	Secondary atom site location: difference Fourier map
*R*[*F*^2^ > 2σ(*F*^2^)] = 0.037	Hydrogen site location: inferred from neighbouring sites
*wR*(*F*^2^) = 0.075	H-atom parameters constrained
*S* = 1.01	*w* = 1/[σ^2^(*F*_o_^2^) + (0.0274*P*)^2^ + 0.3128*P*] where *P* = (*F*_o_^2^ + 2*F*_c_^2^)/3
6628 reflections	(Δ/σ)_max_ = 0.001
307 parameters	Δρ_max_ = 0.36 e Å^−^^3^
0 restraints	Δρ_min_ = −0.28 e Å^−^^3^

### 2-Bromo-1,1,1,3,3,3-hexaisopropyl-2-(triisopropylsilyl)trisilane (II) Special details

**Table:**

Geometry. All esds (except the esd in the dihedral angle between two l.s. planes) are estimated using the full covariance matrix. The cell esds are taken into account individually in the estimation of esds in distances, angles and torsion angles; correlations between esds in cell parameters are only used when they are defined by crystal symmetry. An approximate (isotropic) treatment of cell esds is used for estimating esds involving l.s. planes.
Refinement. All H atoms were positioned geometrically and refined using a riding model with C—H = 0.95–0.99 Å and wAith *U*_iso_(H) = 1.2 (1.5 for methyl groups) times *U*_eq_(C).

### 2-Bromo-1,1,1,3,3,3-hexaisopropyl-2-(triisopropylsilyl)trisilane (II) Fractional atomic coordinates and isotropic or equivalent isotropic displacement parameters (Å^2^)

**Table:**

	*x*	*y*	*z*	*U*_iso_*/*U*_eq_	
Br1	1.06221 (3)	0.19737 (3)	0.24790 (2)	0.02768 (9)	
Si1	0.77723 (8)	0.16573 (6)	0.25116 (3)	0.01926 (16)	
Si2	0.72101 (8)	0.26469 (7)	0.14487 (4)	0.02137 (17)	
Si3	0.67829 (9)	−0.06634 (7)	0.24791 (4)	0.02310 (17)	
Si4	0.74993 (9)	0.27556 (7)	0.36447 (4)	0.02177 (17)	
C1	0.8269 (3)	0.4443 (2)	0.15761 (13)	0.0249 (6)	
H1	0.7549	0.4757	0.1905	0.030*	
C2	1.0018 (3)	0.4866 (3)	0.19270 (15)	0.0349 (7)	
H2A	1.0777	0.4575	0.1626	0.052*	
H2B	0.9962	0.4498	0.2393	0.052*	
H2C	1.0435	0.5794	0.1987	0.052*	
C3	0.8371 (4)	0.5176 (3)	0.08928 (14)	0.0335 (7)	
H3A	0.8753	0.6091	0.1015	0.050*	
H3B	0.7262	0.4931	0.0660	0.050*	
H3C	0.9162	0.4972	0.0570	0.050*	
C4	0.7935 (3)	0.1980 (3)	0.06084 (12)	0.0266 (6)	
H4	0.7464	0.1034	0.0618	0.032*	
C5	0.9832 (3)	0.2301 (3)	0.05683 (14)	0.0371 (7)	
H5A	1.0101	0.1830	0.0161	0.056*	
H5B	1.0268	0.2065	0.1006	0.056*	
H5C	1.0345	0.3214	0.0513	0.056*	
C6	0.7282 (4)	0.2347 (3)	−0.00855 (13)	0.0388 (7)	
H6A	0.7814	0.3257	−0.0146	0.058*	
H6B	0.6067	0.2155	−0.0066	0.058*	
H6C	0.7549	0.1862	−0.0487	0.058*	
C7	0.4836 (3)	0.2270 (2)	0.13810 (14)	0.0287 (6)	
H7	0.4439	0.2136	0.1879	0.034*	
C8	0.4264 (4)	0.3368 (3)	0.11280 (17)	0.0429 (8)	
H8A	0.4536	0.3500	0.0627	0.064*	
H8B	0.4841	0.4146	0.1414	0.064*	
H8C	0.3053	0.3155	0.1182	0.064*	
C9	0.3878 (3)	0.1029 (3)	0.09537 (15)	0.0394 (7)	
H9A	0.2692	0.0772	0.1073	0.059*	
H9B	0.4342	0.0360	0.1073	0.059*	
H9C	0.3991	0.1169	0.0444	0.059*	
C10	0.6700 (3)	−0.1257 (3)	0.15145 (13)	0.0304 (6)	
H10	0.6105	−0.0769	0.1245	0.036*	
C11	0.8446 (4)	−0.0949 (3)	0.11986 (15)	0.0402 (8)	
H11A	0.9038	−0.1481	0.1406	0.060*	
H11B	0.9078	−0.0052	0.1305	0.060*	
H11C	0.8337	−0.1118	0.0683	0.060*	
C12	0.5697 (4)	−0.2671 (3)	0.13666 (15)	0.0423 (8)	
H12A	0.5798	−0.2910	0.0866	0.063*	
H12B	0.4517	−0.2814	0.1470	0.063*	
H12C	0.6138	−0.3189	0.1669	0.063*	
C13	0.4641 (3)	−0.1251 (2)	0.28895 (14)	0.0278 (6)	
H13	0.4741	−0.0754	0.3352	0.033*	
C14	0.3289 (4)	−0.0951 (3)	0.24525 (16)	0.0426 (8)	
H14A	0.3107	−0.1440	0.1997	0.064*	
H14B	0.3651	−0.0041	0.2367	0.064*	
H14C	0.2244	−0.1178	0.2714	0.064*	
C15	0.4016 (4)	−0.2660 (3)	0.30713 (16)	0.0402 (7)	
H15A	0.2967	−0.2836	0.3327	0.060*	
H15B	0.4859	−0.2842	0.3371	0.060*	
H15C	0.3827	−0.3196	0.2632	0.060*	
C16	0.8392 (3)	−0.1171 (2)	0.29934 (14)	0.0292 (6)	
H16	0.9513	−0.0608	0.2850	0.035*	
C17	0.8383 (4)	−0.2537 (3)	0.28398 (15)	0.0383 (7)	
H17A	0.9229	−0.2705	0.3143	0.057*	
H17B	0.8637	−0.2649	0.2340	0.057*	
H17C	0.7277	−0.3129	0.2938	0.057*	
C18	0.8317 (4)	−0.0933 (3)	0.38032 (15)	0.0461 (8)	
H18A	0.7258	−0.1494	0.3976	0.069*	
H18B	0.8392	−0.0047	0.3909	0.069*	
H18C	0.9252	−0.1109	0.4039	0.069*	
C19	0.5967 (3)	0.1584 (2)	0.42272 (13)	0.0290 (6)	
H19	0.6217	0.0766	0.4166	0.035*	
C20	0.4130 (4)	0.1287 (3)	0.40018 (18)	0.0467 (8)	
H20A	0.3450	0.0522	0.4228	0.070*	
H20B	0.4012	0.1145	0.3484	0.070*	
H20C	0.3751	0.2003	0.4149	0.070*	
C21	0.6171 (4)	0.1952 (3)	0.50277 (14)	0.0486 (9)	
H21A	0.5929	0.2749	0.5118	0.073*	
H21B	0.7321	0.2064	0.5185	0.073*	
H21C	0.5392	0.1280	0.5290	0.073*	
C22	0.9557 (3)	0.3395 (2)	0.41777 (13)	0.0286 (6)	
H22	0.9246	0.3756	0.4631	0.034*	
C23	1.0335 (4)	0.2414 (3)	0.44175 (15)	0.0397 (7)	
H23A	1.0703	0.2024	0.4002	0.059*	
H23B	0.9504	0.1757	0.4669	0.059*	
H23C	1.1299	0.2831	0.4736	0.059*	
C24	1.0893 (3)	0.4526 (3)	0.38677 (14)	0.0364 (7)	
H24A	1.1806	0.4887	0.4218	0.055*	
H24B	1.0390	0.5175	0.3750	0.055*	
H24C	1.1334	0.4236	0.3437	0.055*	
C25	0.6831 (3)	0.4212 (2)	0.34999 (13)	0.0258 (6)	
H25	0.7695	0.4766	0.3192	0.031*	
C26	0.6873 (4)	0.5007 (3)	0.41963 (14)	0.0450 (8)	
H26A	0.6597	0.5772	0.4092	0.067*	
H26B	0.7994	0.5251	0.4417	0.067*	
H26C	0.6053	0.4502	0.4522	0.067*	
C27	0.5152 (4)	0.3988 (3)	0.31189 (15)	0.0392 (7)	
H27A	0.4243	0.3572	0.3434	0.059*	
H27B	0.5082	0.3445	0.2688	0.059*	
H27C	0.5050	0.4803	0.2990	0.059*	

### 2-Bromo-1,1,1,3,3,3-hexaisopropyl-2-(triisopropylsilyl)trisilane (II) Atomic displacement parameters (Å^2^)

**Table:**

	*U*^11^	*U*^22^	*U*^33^	*U*^12^	*U*^13^	*U*^23^
Br1	0.02088 (14)	0.03279 (18)	0.02927 (15)	0.00846 (11)	0.00155 (11)	0.00149 (12)
Si1	0.0195 (4)	0.0190 (4)	0.0191 (4)	0.0060 (3)	0.0012 (3)	0.0007 (3)
Si2	0.0215 (4)	0.0220 (4)	0.0204 (4)	0.0066 (3)	0.0003 (3)	0.0017 (3)
Si3	0.0256 (4)	0.0192 (4)	0.0247 (4)	0.0076 (3)	0.0019 (3)	0.0009 (3)
Si4	0.0255 (4)	0.0205 (4)	0.0195 (4)	0.0075 (3)	0.0028 (3)	0.0008 (3)
C1	0.0272 (14)	0.0233 (15)	0.0229 (14)	0.0060 (12)	0.0038 (11)	0.0043 (11)
C2	0.0326 (16)	0.0281 (17)	0.0391 (17)	0.0031 (13)	−0.0053 (13)	0.0016 (13)
C3	0.0416 (18)	0.0255 (16)	0.0336 (16)	0.0103 (13)	0.0054 (13)	0.0078 (13)
C4	0.0325 (15)	0.0280 (16)	0.0199 (13)	0.0110 (12)	−0.0005 (11)	−0.0002 (11)
C5	0.0398 (18)	0.049 (2)	0.0266 (15)	0.0205 (15)	0.0080 (13)	0.0018 (14)
C6	0.0447 (18)	0.047 (2)	0.0228 (15)	0.0122 (15)	−0.0018 (13)	−0.0007 (14)
C7	0.0234 (15)	0.0325 (17)	0.0306 (15)	0.0091 (12)	−0.0023 (12)	0.0033 (13)
C8	0.0280 (16)	0.044 (2)	0.059 (2)	0.0136 (14)	−0.0057 (15)	0.0083 (16)
C9	0.0287 (16)	0.0406 (19)	0.0425 (18)	0.0022 (14)	−0.0059 (13)	0.0037 (14)
C10	0.0392 (17)	0.0255 (16)	0.0284 (15)	0.0138 (13)	0.0009 (13)	−0.0026 (12)
C11	0.053 (2)	0.0409 (19)	0.0339 (16)	0.0245 (15)	0.0101 (15)	0.0006 (14)
C12	0.055 (2)	0.0358 (19)	0.0371 (17)	0.0182 (16)	−0.0091 (15)	−0.0096 (14)
C13	0.0281 (15)	0.0207 (15)	0.0328 (15)	0.0053 (12)	0.0030 (12)	0.0029 (12)
C14	0.0303 (17)	0.044 (2)	0.052 (2)	0.0098 (14)	0.0021 (14)	0.0088 (15)
C15	0.0404 (18)	0.0301 (17)	0.0447 (18)	0.0030 (14)	0.0061 (14)	0.0067 (14)
C16	0.0312 (15)	0.0220 (16)	0.0371 (16)	0.0119 (12)	−0.0010 (12)	0.0045 (12)
C17	0.0431 (18)	0.0310 (18)	0.0456 (18)	0.0180 (14)	0.0007 (15)	0.0072 (14)
C18	0.062 (2)	0.042 (2)	0.0410 (18)	0.0268 (17)	−0.0133 (16)	−0.0041 (15)
C19	0.0364 (16)	0.0218 (16)	0.0287 (15)	0.0086 (12)	0.0091 (12)	0.0038 (12)
C20	0.0369 (18)	0.0338 (19)	0.069 (2)	0.0089 (14)	0.0210 (16)	0.0101 (16)
C21	0.075 (2)	0.036 (2)	0.0324 (17)	0.0132 (17)	0.0229 (16)	0.0069 (14)
C22	0.0327 (16)	0.0310 (17)	0.0212 (14)	0.0097 (13)	−0.0035 (12)	−0.0036 (12)
C23	0.0491 (19)	0.042 (2)	0.0312 (16)	0.0197 (15)	−0.0109 (14)	−0.0033 (14)
C24	0.0348 (17)	0.0362 (18)	0.0322 (16)	0.0041 (14)	−0.0043 (13)	−0.0040 (13)
C25	0.0347 (16)	0.0231 (15)	0.0211 (13)	0.0117 (12)	0.0018 (12)	−0.0006 (11)
C26	0.077 (2)	0.0380 (19)	0.0302 (16)	0.0336 (17)	−0.0023 (16)	−0.0064 (14)
C27	0.0388 (18)	0.0384 (19)	0.0464 (18)	0.0213 (14)	−0.0010 (14)	0.0007 (15)

### 2-Bromo-1,1,1,3,3,3-hexaisopropyl-2-(triisopropylsilyl)trisilane (II) Geometric parameters (Å, º)

**Table:**

Br1—Si1	2.3185 (7)	C12—H12B	0.9800
Si1—Si2	2.4430 (10)	C12—H12C	0.9800
Si1—Si3	2.4448 (10)	C13—C14	1.531 (4)
Si1—Si4	2.4628 (9)	C13—C15	1.541 (4)
Si2—C4	1.908 (2)	C13—H13	1.0000
Si2—C7	1.913 (3)	C14—H14A	0.9800
Si2—C1	1.914 (3)	C14—H14B	0.9800
Si3—C10	1.899 (3)	C14—H14C	0.9800
Si3—C13	1.899 (3)	C15—H15A	0.9800
Si3—C16	1.903 (3)	C15—H15B	0.9800
Si4—C22	1.909 (3)	C15—H15C	0.9800
Si4—C25	1.910 (3)	C16—C17	1.533 (4)
Si4—C19	1.911 (3)	C16—C18	1.543 (4)
C1—C2	1.532 (3)	C16—H16	1.0000
C1—C3	1.543 (4)	C17—H17A	0.9800
C1—H1	1.0000	C17—H17B	0.9800
C2—H2A	0.9800	C17—H17C	0.9800
C2—H2B	0.9800	C18—H18A	0.9800
C2—H2C	0.9800	C18—H18B	0.9800
C3—H3A	0.9800	C18—H18C	0.9800
C3—H3B	0.9800	C19—C20	1.530 (4)
C3—H3C	0.9800	C19—C21	1.539 (4)
C4—C5	1.528 (4)	C19—H19	1.0000
C4—C6	1.536 (4)	C20—H20A	0.9800
C4—H4	1.0000	C20—H20B	0.9800
C5—H5A	0.9800	C20—H20C	0.9800
C5—H5B	0.9800	C21—H21A	0.9800
C5—H5C	0.9800	C21—H21B	0.9800
C6—H6A	0.9800	C21—H21C	0.9800
C6—H6B	0.9800	C22—C23	1.525 (4)
C6—H6C	0.9800	C22—C24	1.538 (4)
C7—C9	1.543 (4)	C22—H22	1.0000
C7—C8	1.544 (4)	C23—H23A	0.9800
C7—H7	1.0000	C23—H23B	0.9800
C8—H8A	0.9800	C23—H23C	0.9800
C8—H8B	0.9800	C24—H24A	0.9800
C8—H8C	0.9800	C24—H24B	0.9800
C9—H9A	0.9800	C24—H24C	0.9800
C9—H9B	0.9800	C25—C27	1.525 (4)
C9—H9C	0.9800	C25—C26	1.543 (3)
C10—C11	1.534 (4)	C25—H25	1.0000
C10—C12	1.541 (4)	C26—H26A	0.9800
C10—H10	1.0000	C26—H26B	0.9800
C11—H11A	0.9800	C26—H26C	0.9800
C11—H11B	0.9800	C27—H27A	0.9800
C11—H11C	0.9800	C27—H27B	0.9800
C12—H12A	0.9800	C27—H27C	0.9800
			
Br1—Si1—Si2	103.77 (3)	C10—C12—H12C	109.5
Br1—Si1—Si3	98.44 (3)	H12A—C12—H12C	109.5
Si2—Si1—Si3	116.55 (3)	H12B—C12—H12C	109.5
Br1—Si1—Si4	102.65 (3)	C14—C13—C15	109.5 (2)
Si2—Si1—Si4	115.02 (4)	C14—C13—Si3	112.65 (19)
Si3—Si1—Si4	116.59 (4)	C15—C13—Si3	116.31 (18)
C4—Si2—C7	108.70 (11)	C14—C13—H13	105.9
C4—Si2—C1	111.49 (12)	C15—C13—H13	105.9
C7—Si2—C1	109.69 (11)	Si3—C13—H13	105.9
C4—Si2—Si1	112.06 (9)	C13—C14—H14A	109.5
C7—Si2—Si1	106.40 (9)	C13—C14—H14B	109.5
C1—Si2—Si1	108.35 (8)	H14A—C14—H14B	109.5
C10—Si3—C13	111.22 (12)	C13—C14—H14C	109.5
C10—Si3—C16	109.44 (12)	H14A—C14—H14C	109.5
C13—Si3—C16	111.50 (12)	H14B—C14—H14C	109.5
C10—Si3—Si1	107.75 (9)	C13—C15—H15A	109.5
C13—Si3—Si1	109.78 (8)	C13—C15—H15B	109.5
C16—Si3—Si1	106.98 (9)	H15A—C15—H15B	109.5
C22—Si4—C25	104.78 (12)	C13—C15—H15C	109.5
C22—Si4—C19	106.42 (12)	H15A—C15—H15C	109.5
C25—Si4—C19	111.59 (12)	H15B—C15—H15C	109.5
C22—Si4—Si1	112.92 (8)	C17—C16—C18	108.9 (2)
C25—Si4—Si1	111.74 (8)	C17—C16—Si3	116.29 (19)
C19—Si4—Si1	109.24 (8)	C18—C16—Si3	112.56 (18)
C2—C1—C3	107.7 (2)	C17—C16—H16	106.1
C2—C1—Si2	115.52 (18)	C18—C16—H16	106.1
C3—C1—Si2	114.54 (18)	Si3—C16—H16	106.1
C2—C1—H1	106.1	C16—C17—H17A	109.5
C3—C1—H1	106.1	C16—C17—H17B	109.5
Si2—C1—H1	106.1	H17A—C17—H17B	109.5
C1—C2—H2A	109.5	C16—C17—H17C	109.5
C1—C2—H2B	109.5	H17A—C17—H17C	109.5
H2A—C2—H2B	109.5	H17B—C17—H17C	109.5
C1—C2—H2C	109.5	C16—C18—H18A	109.5
H2A—C2—H2C	109.5	C16—C18—H18B	109.5
H2B—C2—H2C	109.5	H18A—C18—H18B	109.5
C1—C3—H3A	109.5	C16—C18—H18C	109.5
C1—C3—H3B	109.5	H18A—C18—H18C	109.5
H3A—C3—H3B	109.5	H18B—C18—H18C	109.5
C1—C3—H3C	109.5	C20—C19—C21	109.0 (2)
H3A—C3—H3C	109.5	C20—C19—Si4	113.92 (19)
H3B—C3—H3C	109.5	C21—C19—Si4	114.62 (19)
C5—C4—C6	108.5 (2)	C20—C19—H19	106.2
C5—C4—Si2	114.13 (17)	C21—C19—H19	106.2
C6—C4—Si2	114.24 (19)	Si4—C19—H19	106.2
C5—C4—H4	106.5	C19—C20—H20A	109.5
C6—C4—H4	106.5	C19—C20—H20B	109.5
Si2—C4—H4	106.5	H20A—C20—H20B	109.5
C4—C5—H5A	109.5	C19—C20—H20C	109.5
C4—C5—H5B	109.5	H20A—C20—H20C	109.5
H5A—C5—H5B	109.5	H20B—C20—H20C	109.5
C4—C5—H5C	109.5	C19—C21—H21A	109.5
H5A—C5—H5C	109.5	C19—C21—H21B	109.5
H5B—C5—H5C	109.5	H21A—C21—H21B	109.5
C4—C6—H6A	109.5	C19—C21—H21C	109.5
C4—C6—H6B	109.5	H21A—C21—H21C	109.5
H6A—C6—H6B	109.5	H21B—C21—H21C	109.5
C4—C6—H6C	109.5	C23—C22—C24	110.6 (2)
H6A—C6—H6C	109.5	C23—C22—Si4	116.47 (19)
H6B—C6—H6C	109.5	C24—C22—Si4	115.61 (18)
C9—C7—C8	109.7 (2)	C23—C22—H22	104.1
C9—C7—Si2	115.43 (18)	C24—C22—H22	104.1
C8—C7—Si2	114.49 (19)	Si4—C22—H22	104.1
C9—C7—H7	105.4	C22—C23—H23A	109.5
C8—C7—H7	105.4	C22—C23—H23B	109.5
Si2—C7—H7	105.4	H23A—C23—H23B	109.5
C7—C8—H8A	109.5	C22—C23—H23C	109.5
C7—C8—H8B	109.5	H23A—C23—H23C	109.5
H8A—C8—H8B	109.5	H23B—C23—H23C	109.5
C7—C8—H8C	109.5	C22—C24—H24A	109.5
H8A—C8—H8C	109.5	C22—C24—H24B	109.5
H8B—C8—H8C	109.5	H24A—C24—H24B	109.5
C7—C9—H9A	109.5	C22—C24—H24C	109.5
C7—C9—H9B	109.5	H24A—C24—H24C	109.5
H9A—C9—H9B	109.5	H24B—C24—H24C	109.5
C7—C9—H9C	109.5	C27—C25—C26	108.5 (2)
H9A—C9—H9C	109.5	C27—C25—Si4	117.05 (18)
H9B—C9—H9C	109.5	C26—C25—Si4	112.35 (18)
C11—C10—C12	110.3 (2)	C27—C25—H25	106.0
C11—C10—Si3	112.34 (19)	C26—C25—H25	106.0
C12—C10—Si3	115.08 (19)	Si4—C25—H25	106.0
C11—C10—H10	106.1	C25—C26—H26A	109.5
C12—C10—H10	106.1	C25—C26—H26B	109.5
Si3—C10—H10	106.1	H26A—C26—H26B	109.5
C10—C11—H11A	109.5	C25—C26—H26C	109.5
C10—C11—H11B	109.5	H26A—C26—H26C	109.5
H11A—C11—H11B	109.5	H26B—C26—H26C	109.5
C10—C11—H11C	109.5	C25—C27—H27A	109.5
H11A—C11—H11C	109.5	C25—C27—H27B	109.5
H11B—C11—H11C	109.5	H27A—C27—H27B	109.5
C10—C12—H12A	109.5	C25—C27—H27C	109.5
C10—C12—H12B	109.5	H27A—C27—H27C	109.5
H12A—C12—H12B	109.5	H27B—C27—H27C	109.5
			
C13—Si3—C10—C11	−172.26 (19)	C10—Si3—C13—C14	−52.9 (2)
C16—Si3—C10—C11	−48.6 (2)	C16—Si3—C13—C14	−175.32 (19)
Si1—Si3—C10—C11	67.4 (2)	Si1—Si3—C13—C14	66.3 (2)
C13—Si3—C10—C12	−44.9 (2)	C10—Si3—C13—C15	74.7 (2)
C16—Si3—C10—C12	78.8 (2)	C16—Si3—C13—C15	−47.8 (2)
Si1—Si3—C10—C12	−165.25 (18)	Si1—Si3—C13—C15	−166.17 (17)

### 2-Bromo-1,1,1,3,3,3-hexaisopropyl-2-(triisopropylsilyl)trisilane (II) Hydrogen-bond geometry (Å, º)

**Table:**

*D*—H···*A*	*D*—H	H···*A*	*D*···*A*	*D*—H···*A*
C5—H5*B*···Br1	0.98	2.80	3.711 (3)	155
C16—H16···Br1	1.00	2.84	3.584 (3)	131
C23—H23*A*···Br1	0.98	2.87	3.685 (3)	142
C24—H24*C*···Br1	0.98	2.93	3.726 (3)	139
